# Supplementary figures and images for: Defining the proteolytic landscape during enterovirus infection
Source: PLoS Pathog. 2020 Sep 30;16(9):e1008927. doi: 10.1371/journal.ppat.1008927 (PMC7549765; doi:10.1371/journal.ppat.1008927)

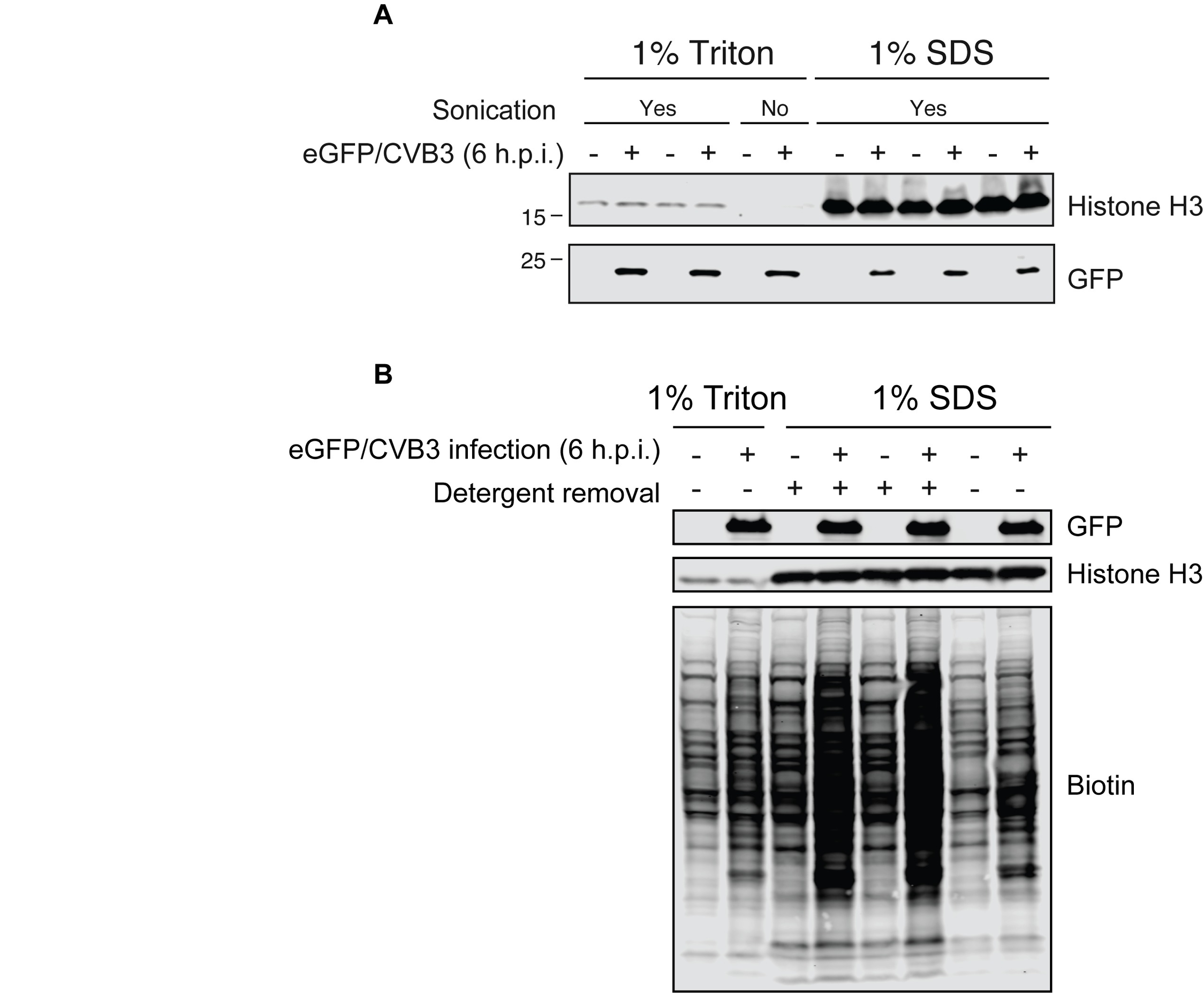

Supplement: S1 Fig — (A) SDS lysis yielded better results than Triton-X lysis. HeLa cells infected with eGFP/CVB3 were lysed in either 1% Triton-X or 1% SDS. The experiment was performed in triplicate. All SDS lysates were sonicated, while out of the three Triton-X lysates, two were sonicated and one was left unsonicated. The western blot was performed with anti-histone H3 and anti-GFP antibody. (B) SDS depletion before labeling was necessary for subtiligase reaction. The HeLa cell lysates were prepared with either 1% Triton-X (single) or 1% SDS (triplicate). Of the three SDS lysates, two were subjected to SDS removal before subtiligase labeling while one was labeled in the presence of SDS. The Triton-X lysate contained the detergent during labeling. The labeling efficiency was determined by western blot with streptavidin. In parallel, the western blot for histone H3 and GFP was also performed. (TIF) [file ppat.1008927.s001.tif]

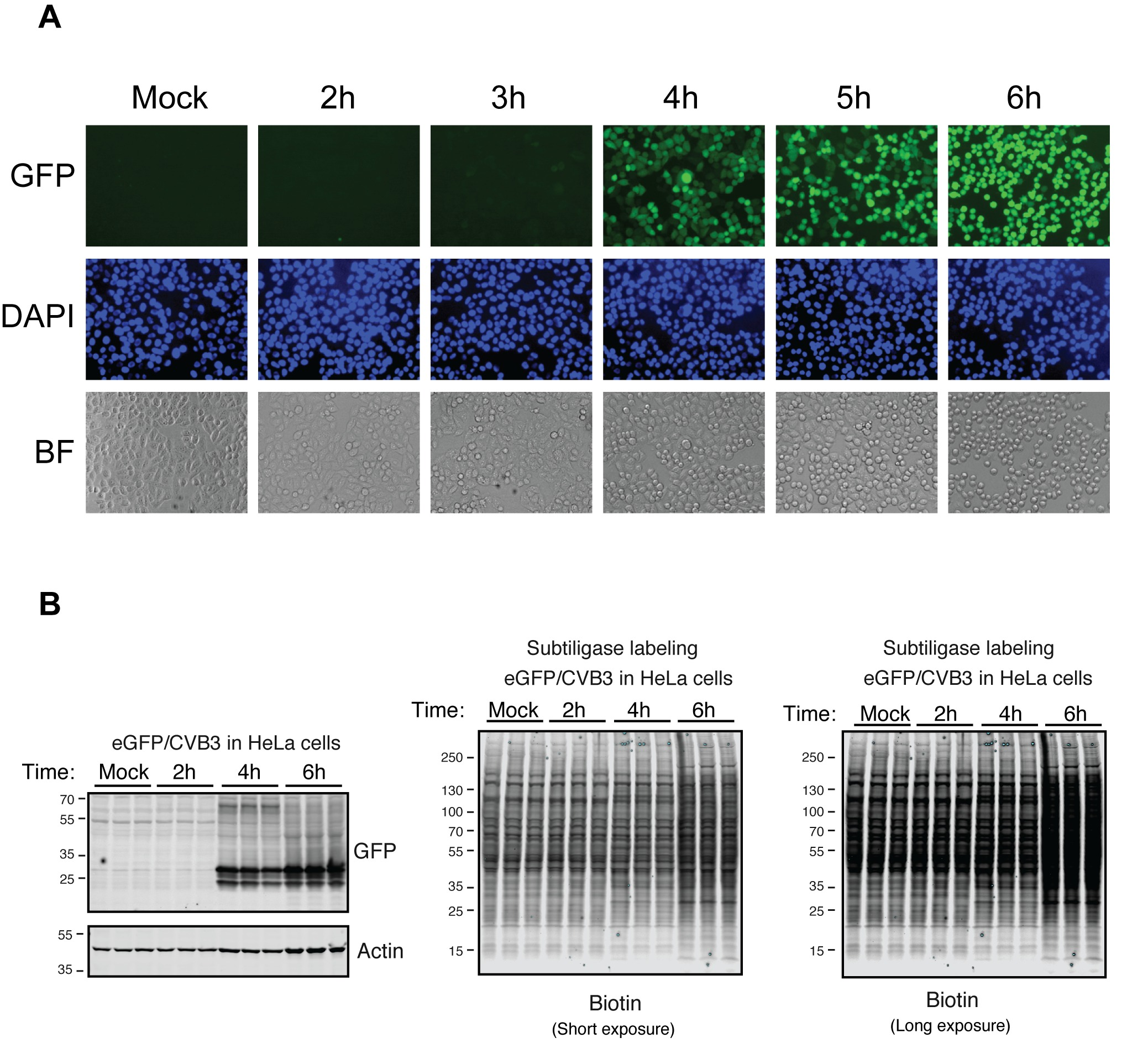

Supplement: S2 Fig — (A) HeLa cells were infected with eGFP/CVB3, and the infection was monitored by GFP detection at the indicated times. DAPI was used for nuclear staining. The bright field (BF) images show progressive cell rounding with infection. (B) HeLa cells infected with eGFP/CVB3 for 2, 4, or 6h, or left uninfected, were lysed in 1% SDS. The lysates were then depleted of SDS and either subjected to western blot to detect GFP and actin (left panel) or incubated with subtiligase and biotinylated peptide. The labeling efficiency was determined by western blot with streptavidin. The two different exposure times are shown. (TIF) [file ppat.1008927.s002.tif]

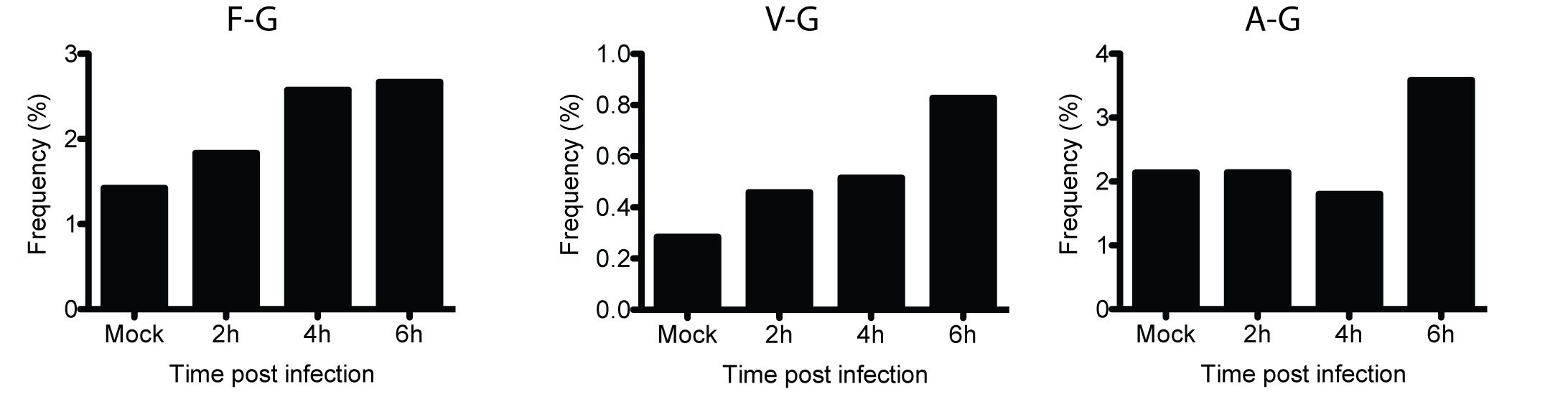

Supplement: S3 Fig — The CVB3 2Apro preferably cleaves proteins at Y-G, T-G, F-G, V-G, and A-G pairs. We calculated the frequency of these cleavages at different times post-infection. The results of Y-G and T-G are shown in Fig 1D, while the rest are shown here. (TIF) [file ppat.1008927.s003.tif]

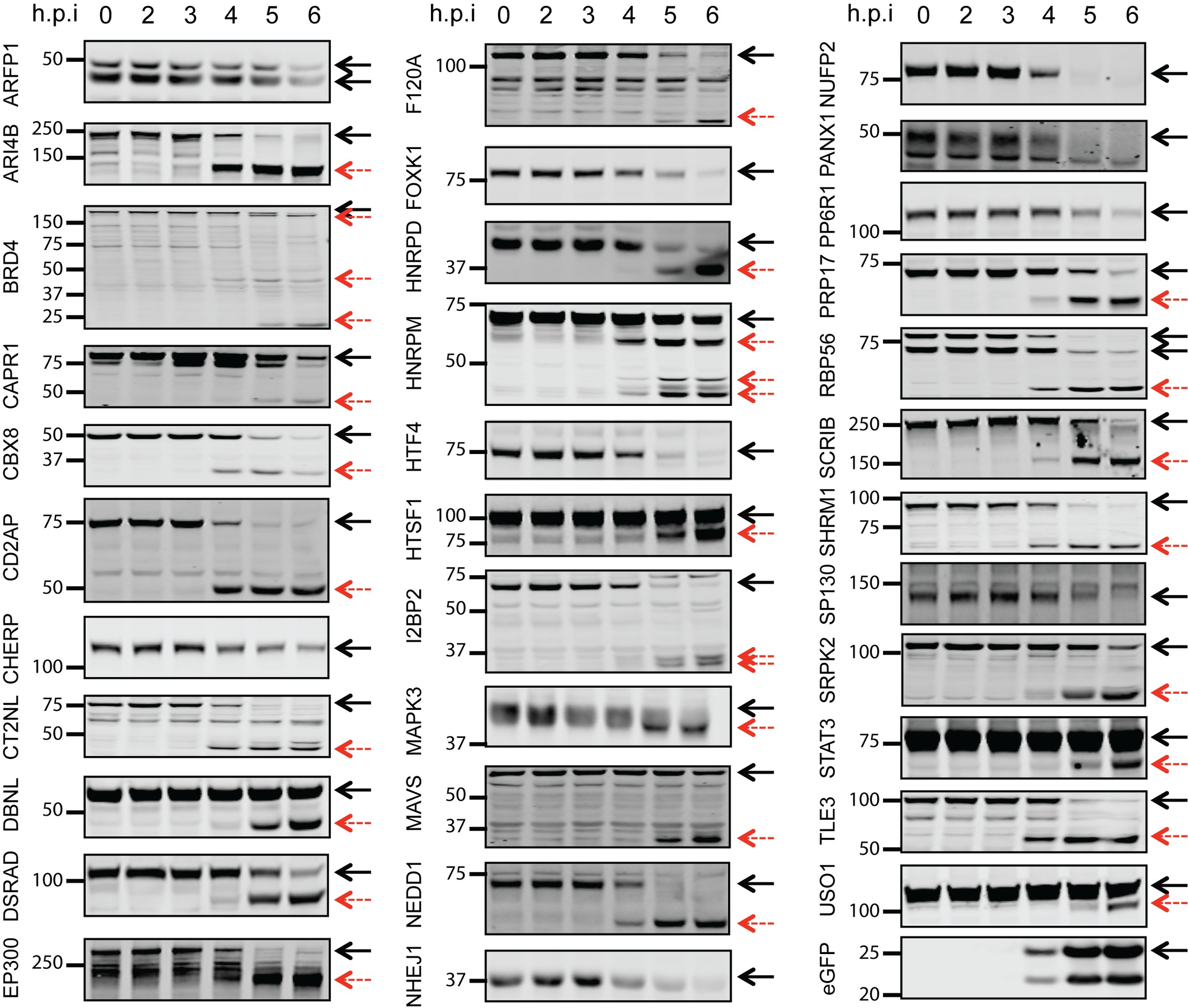

Supplement: S4 Fig — HeLa cells were infected with eGFP/CVB3 and lysed at 0, 2, 3, 4, 5, and 6 h.p.i. followed by western blot analysis of the indicated proteins. An equal amount of total protein, as quantified by the BCA assay, was loaded for each time point. The GFP expression was used to monitor the progression of infection (last panel). The black solid arrows indicate the full-length protein, while the cleavage products are shown with red dotted arrows. Some of the western blot images are shown in Fig 2. (TIF) [file ppat.1008927.s004.tif]

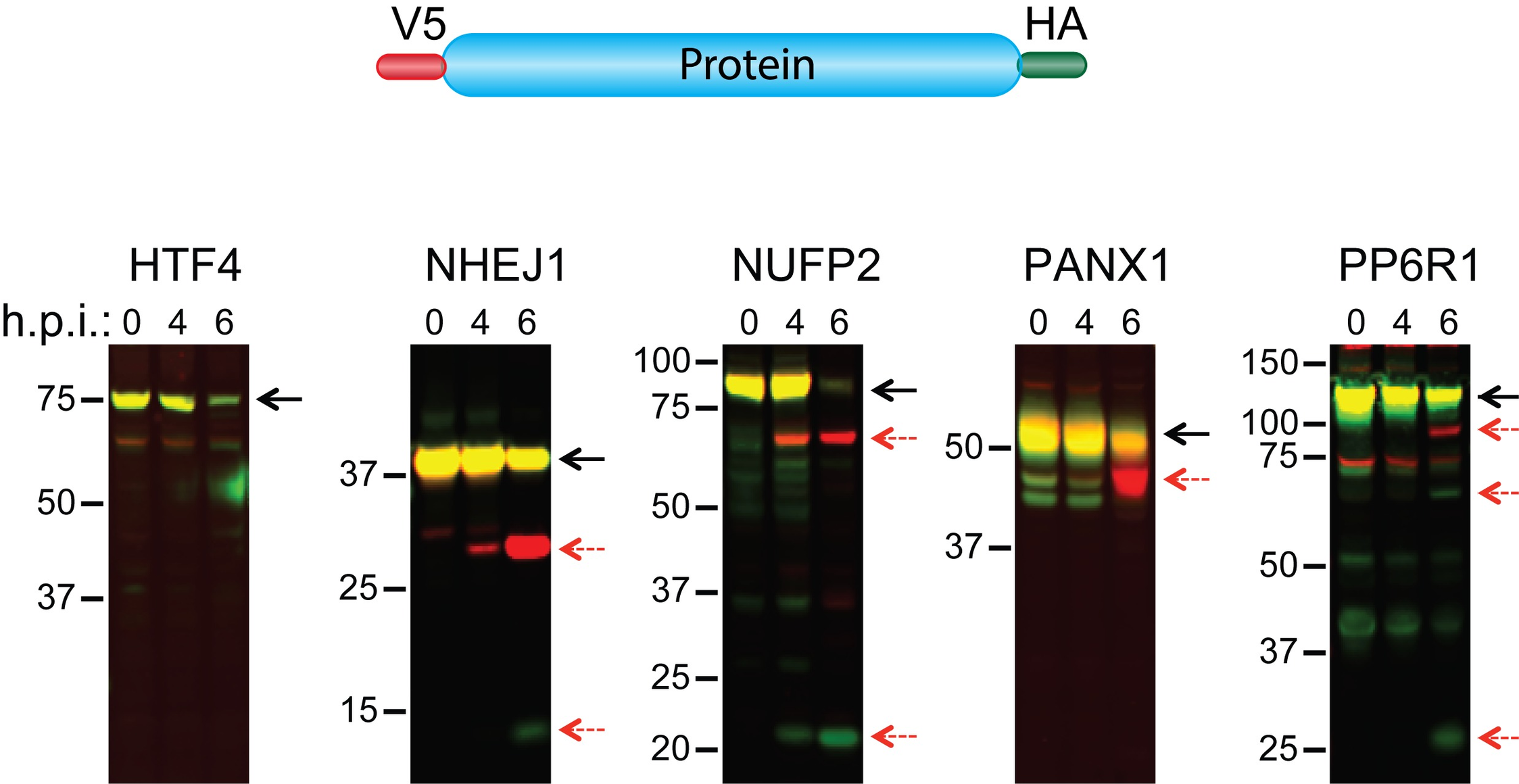

Supplement: S5 Fig — (Top panel) General schematics of protein tagging with V5 at the N-terminal end and HA at the C-terminal end. (Bottom panel) HeLa cells transduced to stably express the indicated doubly tagged proteins were infected with eGFP/CVB3 for 4h or 6h, or left uninfected followed by detection of cleavage by western blot. The blots were probed with mouse anti-V5 and rabbit anti-HA antibodies and detected with IRDye 680RD goat anti-mouse IgG (red channel) and IRDye 800CW goat anti-rabbit IgG (green channel). The full-length protein is indicated with black solid arrows, while the cleavage products are indicated with red dotted arrows. (TIF) [file ppat.1008927.s005.tif]

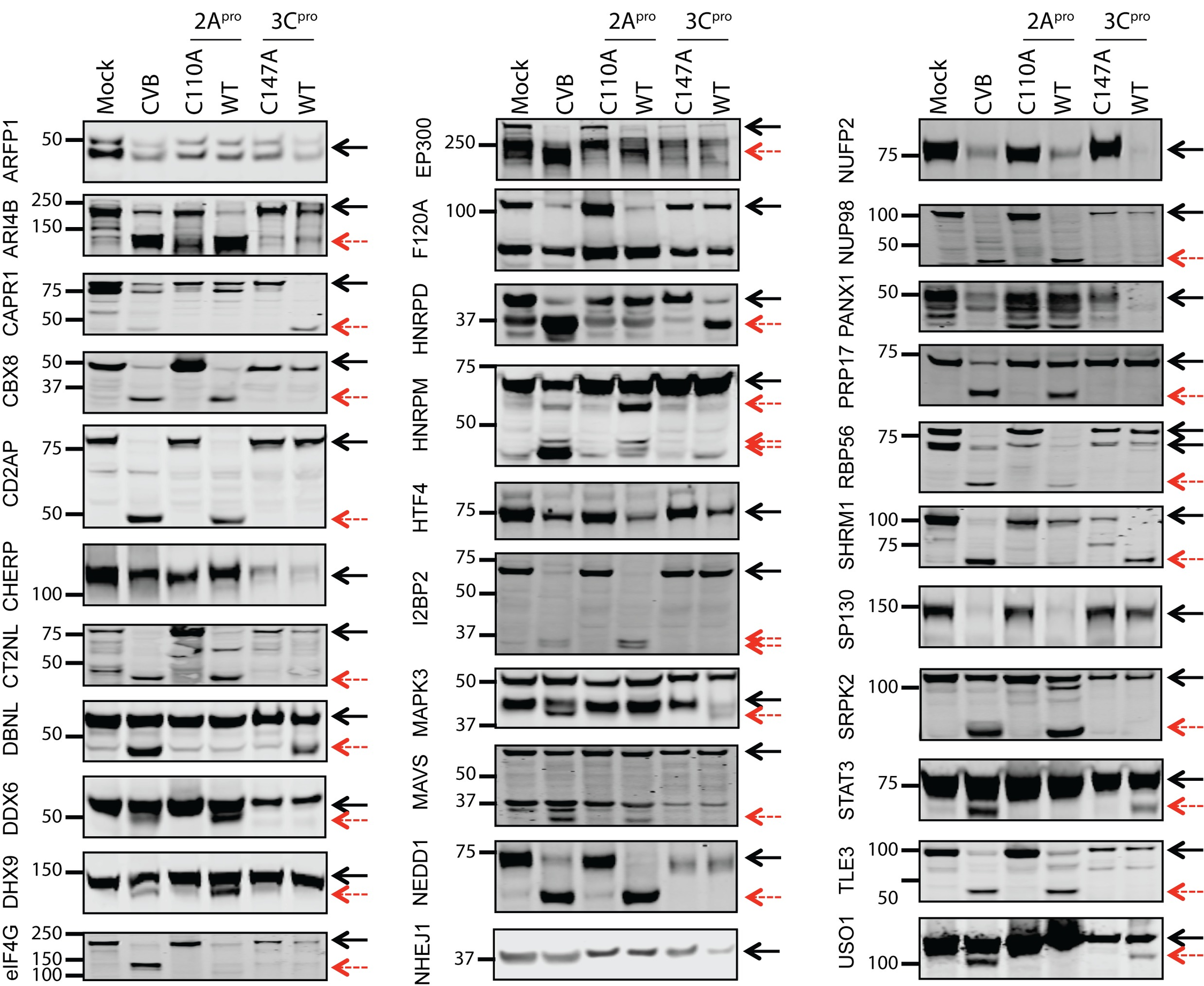

Supplement: S6 Fig — HeLa cell lysates (200 μg protein) were incubated with CVB3 2Apro or its catalytically inactive mutant C110A (1 μg), or CVB3 3Cpro or its catalytically inactive mutant C147A (100 μg) at 37°C for 3h and analyzed by western blot. Lysates from the uninfected and eGFP/CVB3-infected HeLa cells were included as positive controls. The full-length protein is indicated with black solid arrows, while the cleavage products are indicated with red dotted arrows. Some of the western blot images are shown in Fig 3. (TIF) [file ppat.1008927.s006.tif]

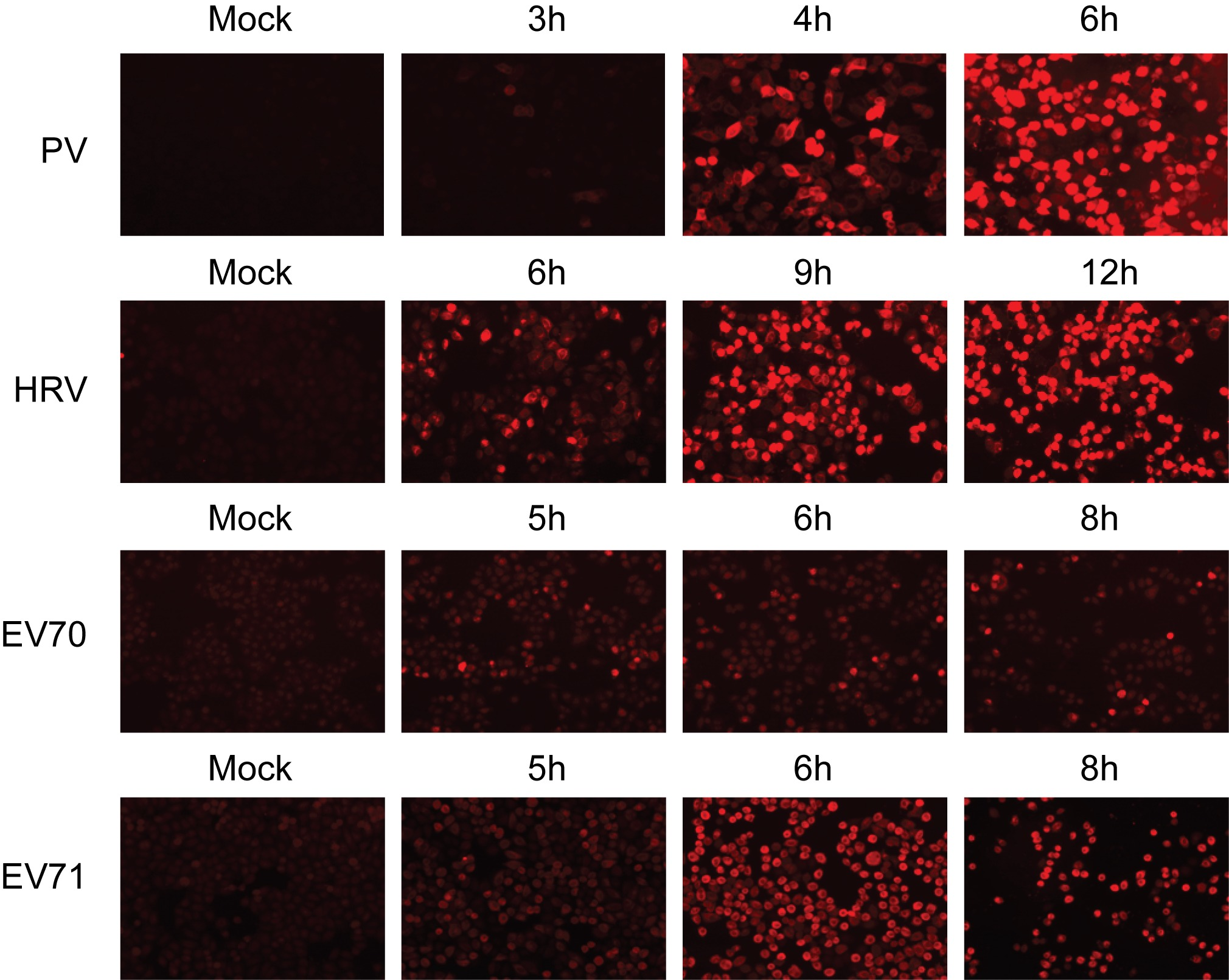

Supplement: S7 Fig — The cells infected with PV (poliovirus), HRV (human rhinovirus A16), EV70 (enterovirus D-70) and EV71 (enterovirus A-71) were fixed in 4% PFA at the indicated times post-infection and analyzed for the expression of viral capsid proteins using antibodies specific to each virus (Material and Methods). (TIF) [file ppat.1008927.s007.tif]

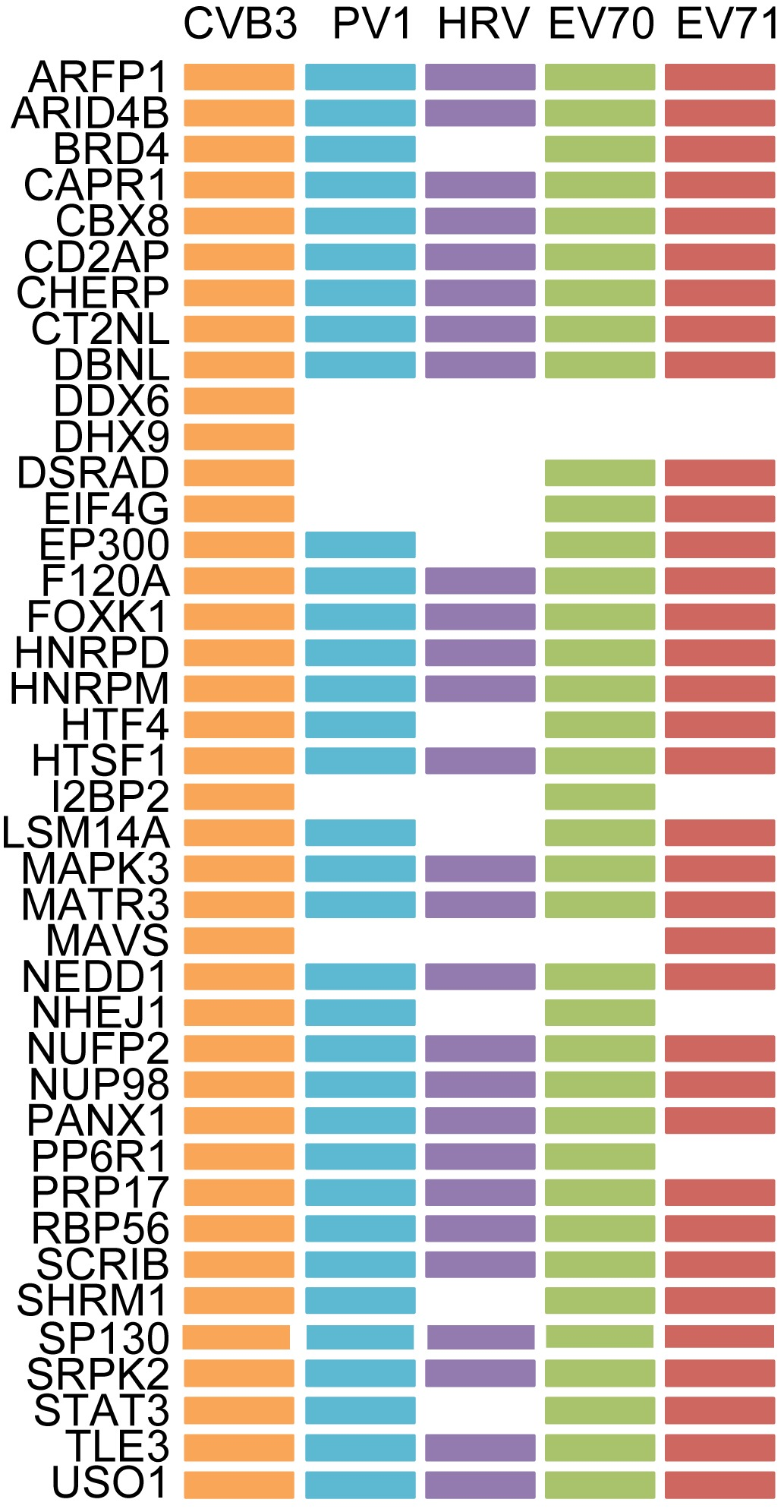

Supplement: S8 Fig — The list of proteins targeted for cleavage in eGFP/CVB3-infected HeLa cells were checked for their presence in the proteomics dataset of the indicated viruses. The tiles, indicating the detection of the proteins shown on left, are color-coded for visual clarity. (TIF) [file ppat.1008927.s008.tif]

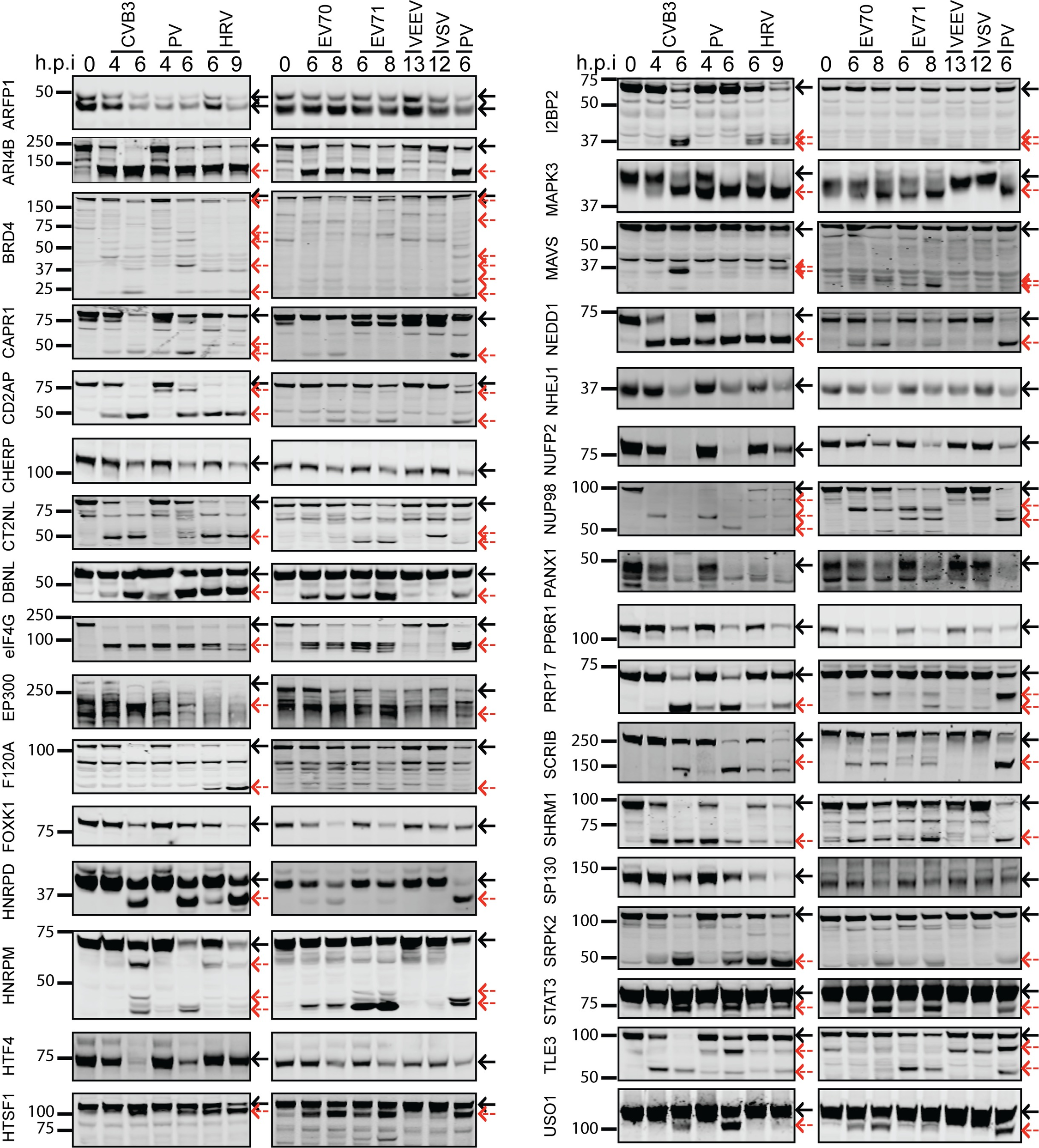

Supplement: S9 Fig — HeLa cells infected with CVB3 (eGFP/CVB3), PV (poliovirus type 1), HRV (human rhinovirus A16), EV70 (enterovirus D-70), EV71 (enterovirus A-71), VEEV (Venezuelan equine encephalitis virus) or VSV (vesicular stomatitis virus) were lysed at the indicated times, and equal amounts of total protein were subjected to western blot with the indicated antibodies. The black solid and red dotted arrows indicate the full-length protein and the cleavage products, respectively. Some of the western blot images are shown in Fig 5. (TIF) [file ppat.1008927.s009.tif]

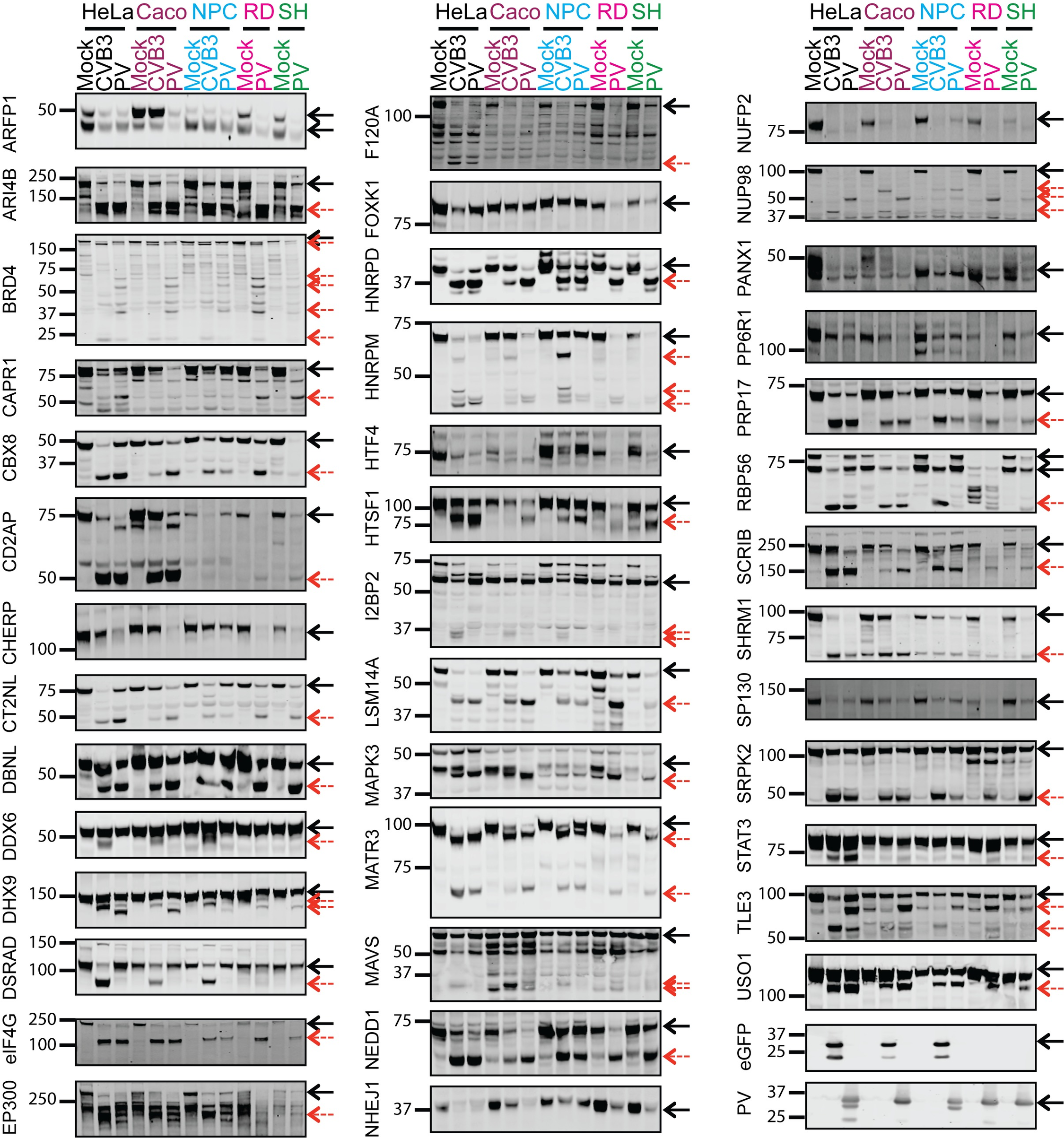

Supplement: S10 Fig — HeLa (cervical epithelial) cells infected with eGFP/CVB3 and PV for 6h, Caco-2 (intestinal epithelial) cells infected with eGFP/CVB3 and PV for 8h, NPC (neural progenitor cells) infected with eGFP/CVB3 and PV for 8h, RD (rhabdomyosarcoma) cells and SK-N-SH (brain epithelial) cells infected with PV for 8h were analyzed for the cleavage of indicated proteins. The full-length protein is shown with black solid arrows, while the cleavage products with red dotted arrows. (TIF) [file ppat.1008927.s010.tif]

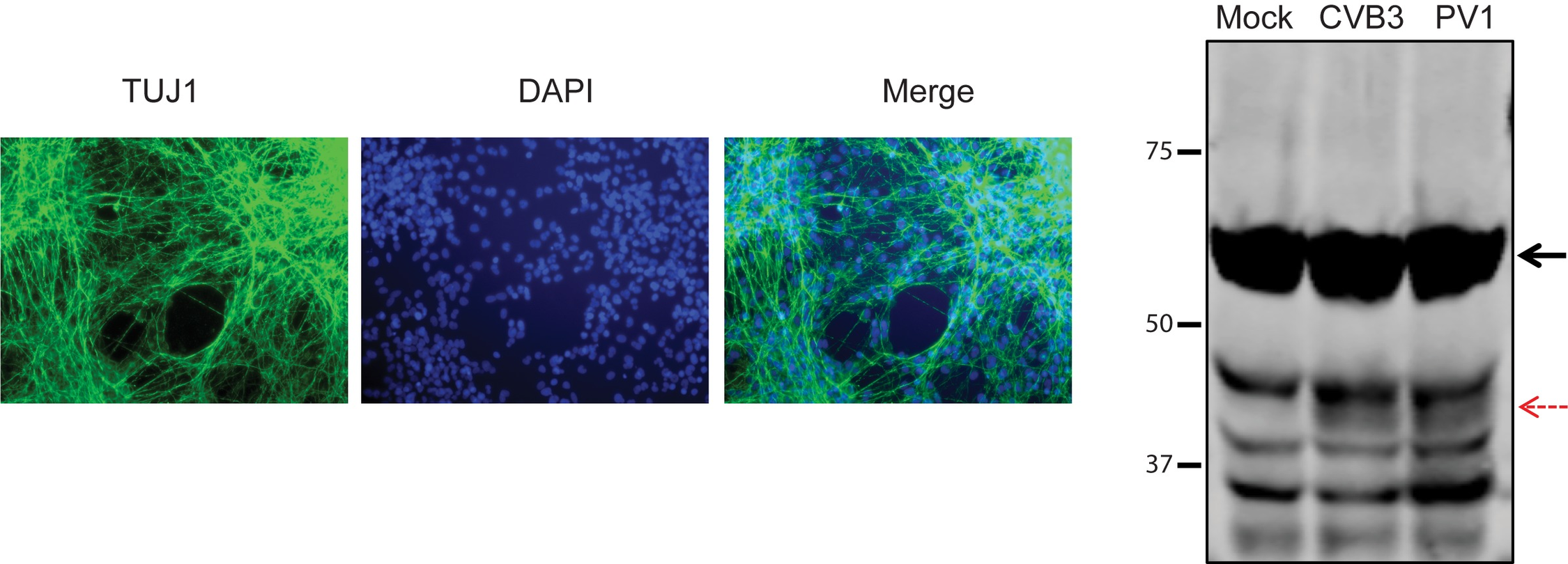

Supplement: S11 Fig — Immunofluorescence images: embryonic stem cell-derived neurons were stained for TUJ1, a neuron-specific protein (left panel), and with DAPI as a nuclear stain (middle panel). The right panel shows the merged image. Western blot: The neurons were infected with eGFP/CVB3 or PV for 7h followed by western blot with anti-LSM14A antibody. The solid black arrow shows the full-length protein while the red dotted arrow indicates the C-terminal cleavage product. (TIF) [file ppat.1008927.s011.tif]

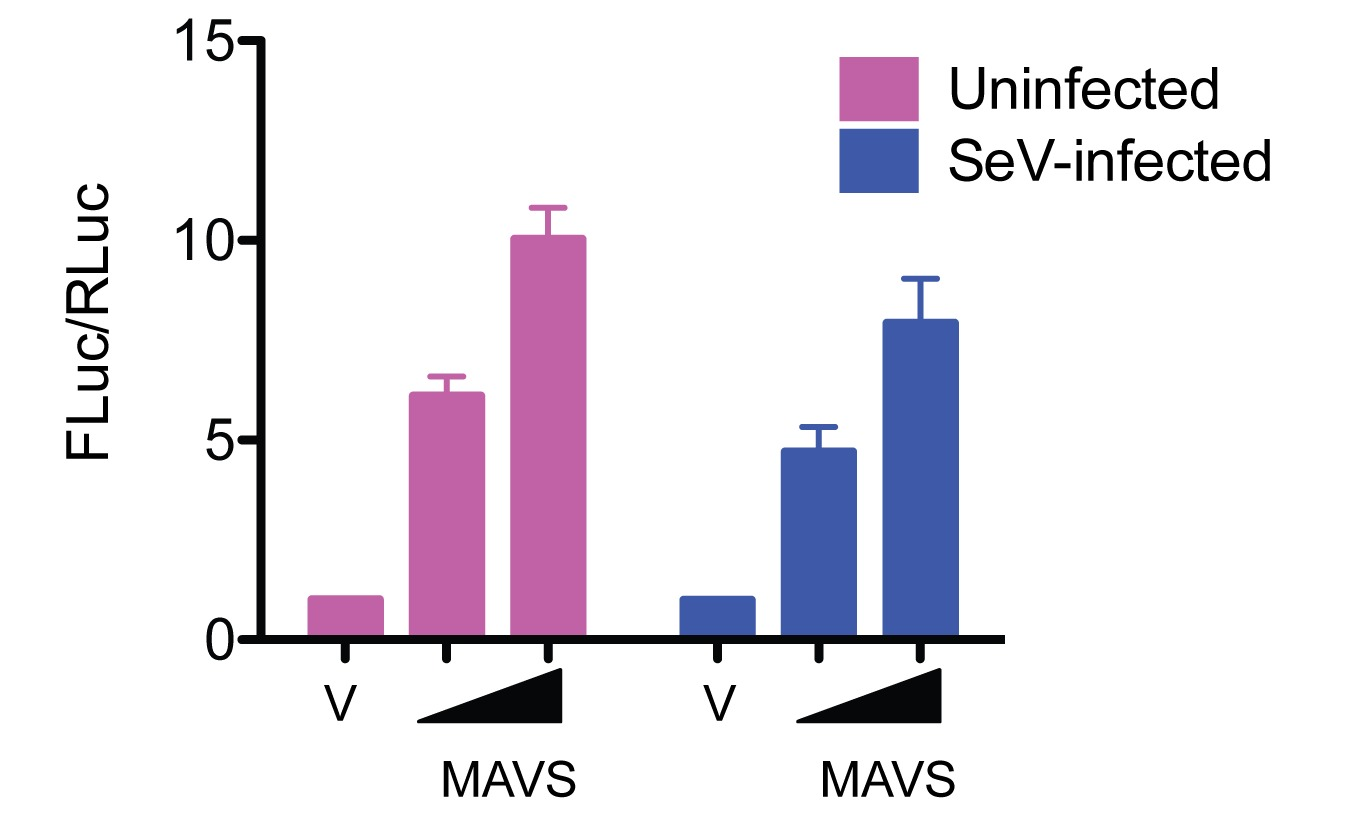

Supplement: S12 Fig — 293T cells, seeded into 48-well plates at a density of 30,000 cells/well, were transfected with the ISRE-FLuc and RL-RLuc reporter plasmids along with GFP plasmid (vector) or increasing concentrations of MAVS plasmid (6.25 and 12.5 ng/well). Twenty-four hours later, the cells were either left uninfected or infected with SeV for 24h followed by the reporter assays as described in Fig 7A. V, vector. (TIF) [file ppat.1008927.s012.tif]

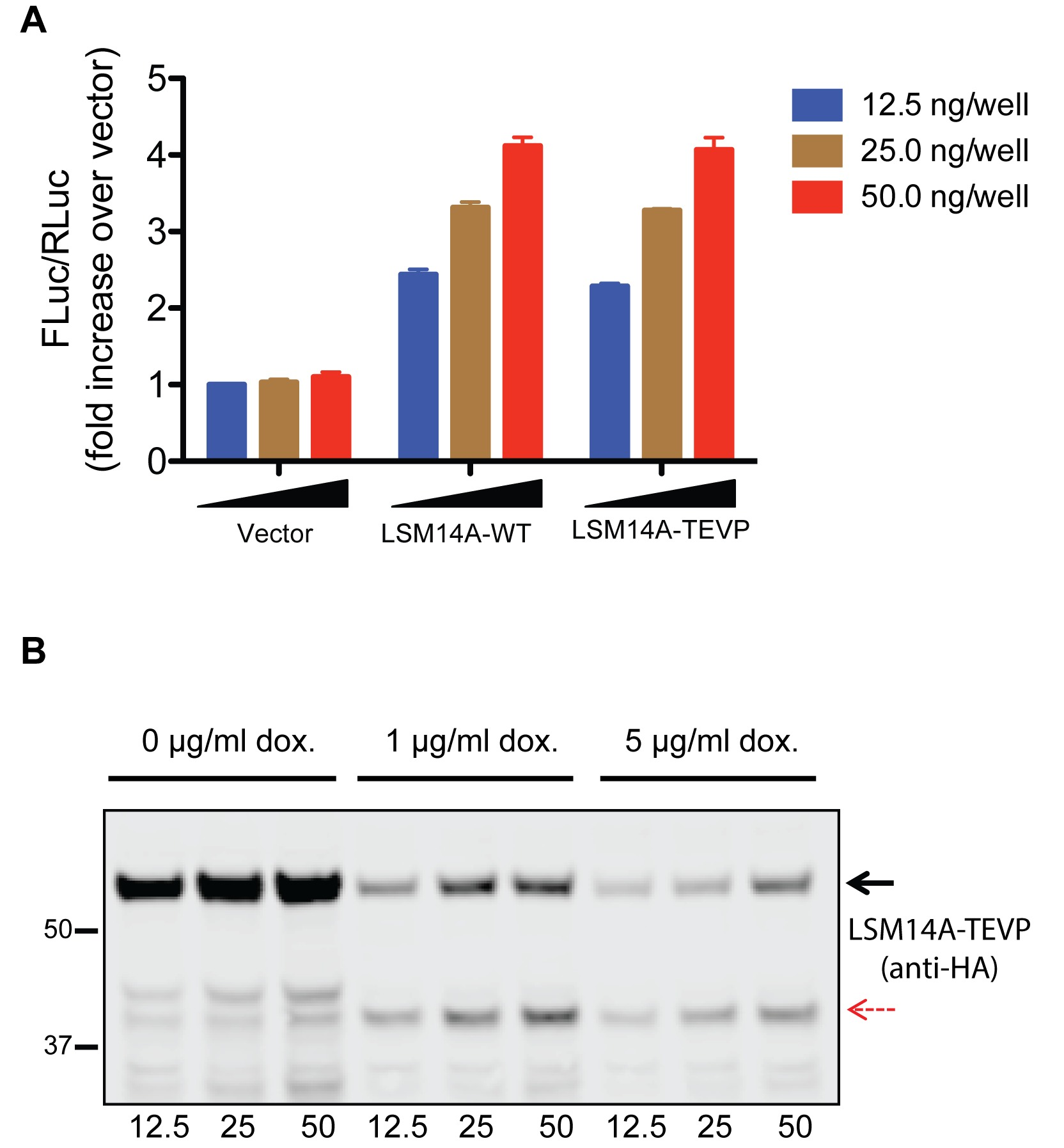

Supplement: S13 Fig — (A) 293T cells were transfected with the indicated concentrations of the vector, LSM14A-WT, or LSM14A-TEVP plasmids and infected with SeV. The reporter assay was performed as described in Fig 7A. (B) The 293T cells containing dox-inducible TEVP were transfected with the increasing amount of the V5-LSM14A-TEVP-HA plasmid and the TEVP expression was induced using the indicated concentrations of doxycycline. The LSM14A cleavage was monitored by western blot with anti-HA antibodies. The small amount of cleavage product seen in the untreated cells is reflective of the leakiness of the dox promoter. (TIF) [file ppat.1008927.s013.tif]
