# Supplementary material for: Defining the proteolytic landscape during enterovirus infection
Source: PLoS Pathog. 2020 Sep 30;16(9):e1008927. doi: 10.1371/journal.ppat.1008927 (PMC7549765; doi:10.1371/journal.ppat.1008927)
Supplement: Attachment — Submitted filename: Rice.pdf [file ppat.1008927.s016.pdf]

In this manuscript, Saeed *et al*. identify sites of host protein cleavage upon enterovirus infection, validate a number of these sites orthogonally, and examine one proteolytic substrate in more detail. It is a substantive and thorough piece of work and perhaps long overdue for the field. It is worthy of publication. None of the following comments detract measurably from this conclusion.

The study raises interesting future questions about how 2A site specificity is achieved. It is also interesting to consider how infection may modulate the cell's natural peptidomic landscape via, for example, the TAP transporters (the MS portion of the study does not seem to require neo-N-termini to represent a simple bisection, or for peptides to be intracellular).

The initial portion of the study, namely the N-terminomics of CVB-3-infected HeLa extract via peptide ligation to unblocked N-termini, was likely challenging to perfect despite optimization: Here, the presence of a substantial unmodified proteome, even after the enrichment steps, is suggested by the setting of Abu as a variable modification in order to achieve acceptable FDR. In future work, this could perhaps be addressed by implementing an uninfected/infected isotope split. In this regard (P.6/Line 19), it is not clear what is meant by "multiplexed samples" since it appears they were not multiplexed according to M&M.

The authors identify unique human targets from single peptides, and the latter would need to be of sufficient length and quality (of fragmentation spectra) to evince unique matches in the human proteome. In this regard, it is not clear whether a peptide length filter was imposed: Parental spectra scanned from a low m/z of 140 Th, sufficiently low to identify Abu-tagged peptides just 2 aa in length. The fragmentation method (presumably HCD, since it was a Q-E?) does not seem to be specified. The authors identify cleavage sites from the downstream portion of the cleavage site only. Albeit unlikely, is there an assumption data do not include secondary sites from exopeptidase nibbling?

<u>*In the following comments, note that the first digit of all line numbers was cut off on my pdf. Also, the supp data did not seem to be available for review (at least in the links/logins I could find).*</u> The authors fund these oversights by the journal.

P.8/Line x72: Were the known protein targets confirmed at the level of target site within the protein?

P.9 Line x80 vs. line x84: Was it 81 or 90 proteins?

P.19/Line x91: How do the authors suppose that enteroviral proteases target intercellular junctions?

In Fig. 2A, might virus-induced loss of function/gain of function correspond to quantitative cleavage/incomplete cleavage respectively? Is it clear why cleavages can be detected prior to the first appearance of eGFP?

P.28/line 601: Induction temperature seems missing.

P.29 line 623: 150 microG/microL -> 15 microG/microL

<u>LC-MS/MS section of M&M</u> should specify nanocolumn dimensions/packing/details, along with high m/z (scan range) and fragmentation method. "tandem MS experiments" -> "Tandem MS".

The authors could have perhaps included a sentence, somewhere, on the known functions of p-bodies. (e.g. in cytoplasmic mRNA turnover).

<u>Wording</u>:

P.4/Line x78 ("2A is only responsible for its own cleavage from the upstream structural protein") should perhaps be re-worded given 2A's diverse set of cellular substrates.

P.7 Lines x34/x35: "Intensity of labeling" could perhaps be replaced with "Diversity of labeling".
P.3/Line x66: Close bracket.
P.2/Line x44: Add "a". P.9/Line 81: were -> are.

P.14/Line x99: “Showed” -> “indicated”
P.17/line x47 “viruses” -> “enteroviruses” (since VEEV & SeV seem not to).
P.17/line x51 “Novel proteins” -> “novel substrates”.
P.18/Line x70 “Two C-terminal domains” -> “Two domains C-terminal to the unstructured region”.
