# Supplementary material for: Defining the proteolytic landscape during enterovirus infection
Source: PLoS Pathog. 2020 Sep 30;16(9):e1008927. doi: 10.1371/journal.ppat.1008927 (PMC7549765; doi:10.1371/journal.ppat.1008927)
Supplement: Attachment — Submitted filename: Saeed et al., Rebuttal letter_MS.pdf [file ppat.1008927.s017.pdf]

August 3, 2020

Dear Bert,

Many thanks for sending our paper out for review. Thanks also to the referees for their critiques and suggestions. We now return a revised manuscript and our point-by-point response to the reviewer's comments. Below are copied the referees' comments (in blue) followed by our responses and how the points have been addressed. Please note that the page and line numbers provided in the rebuttal letter are based on the clean version of the manuscript.

We note that reviewer #1 requested a number of additional experiments on one of the viral protease targets, LSM14A. As noted below, some of these data were already included in the supplementary information, which was apparently not accessible to the reviewer. In any case, our intent was to point out one example of a new viral protease target, where cleavage could result in a gain or loss of function (which our data indicate). Since the bulk of the paper presents methods broadly applicable to virus infections, in general, rather than a detailed mechanistic dissection of one target, we have provided some additional data and softened statements accordingly, as requested, but feel that the entire suite of requested LSM14A experiments is far beyond the scope of this paper. This will be the subject of future work when our laboratories are able to resume normal operations.

Thanks again for considering this work for *PLoS Pathogens*.

Kind regards, Charlie and Mohsan

Referee #1 (Remarks to the Author):

The manuscript by Saeed et al examines the host proteolytic network in enterovirus infected cells using an unbiased positive-selection N-terminomics approach. Specifically, this approach (developed by Jim Wells's group) involves appending a biotin-peptide to neo-N-terminal proteins using subtiligase. Although this method has been used successfully to identify cleaved proteins under stress conditions and in in vitro studies using a viral protease (flavivirus), this is the first study to apply this approach in virus-infected cells and (to my knowledge) is the first N-terminomics approach in virus infected cells. The study is comprehensive, applying this approach in five different enterovirus infections in a time dependent manner in order to capture temporal cleavage events. Through extensive (impressive) immunoblotting, they validate 33 novel targets in infected cells and in in vitro cleavage assays using cell lysates. Not surprisingly, they identify targets with a wide range of biological functions and reveal common targets between all enteroviruses and targets that are specific to a subset of enterovirus

infections as well as those that are 3C and 2A protease specific. Overall, this part of the study is impressive, providing the first comprehensive view of the cleaved proteome in enterovirus infected cells with extensive validation and analysis of the cleavage sites. Further, this study shows the power and utility of N-terminomic approaches in revealing novel virus host interactions. Also a note, the authors optimized the subtiligase approach to use 10 fold less lysate (~3 mg), which was a barrier in previous protocols.
In order to show the functional significance of a 2Aprotease-cleaved target, they chose to follow up with LSM14A, a P bodies factor that has been implicated in innate immune signaling in SeV and HSV infection. Using overexpression studies and KO cell lines, the authors show that LSM14A expression activates ISRE transcription under SeV infection (not in mock cells) and that the cleaved N or C term fragments do no induce. Using a clever approach, they replace the 2A cleavage site within a tagged LSM14A with a TEV cleavage site and show that induced expression of TEV protease in cells led to loss of ISRE activation. Although these results implicate LSM14A in innate immune signaling, unfortunately, the exact role of LSM14A in this signaling pathway and how cleavage would affect this is not clear. Finally, the importance of cleavage of LSM14A in enterovirus infected cells could not be shown.
In sum, this a comprehensive study that identifies novel cleaved proteins in infected cells using an innovative N-terminomics approach. The identification of these cleaved proteins should will undoubtedly be seminal for future studies in understanding virus host interactions in enterovirus infections. However, there are several outstanding issues with LSM14A that need to be addressed.

SPECIFIC COMMENTS:

1) The functional significance of LSM14A cleavage was examined in SeV infection and following ISRE activation. In Fig 7A, it is not shown that LSM14A containing the TEV protease site is cleaved by TEV. It is important to show that LSM14A is cleaved and the protein products are similar to that in enterovirus infected cells.

It seems that a glitch in the system precluded the availability of supplementary figures to some of the reviewers. We agree that it is important to test if the LSM14A protein engineered to contain the TEV protease cleavage site (LSM14A-TEVP) was indeed cleaved by the protease, and in fact, we had included this data in the Figure S13B. When LSM14A-TEVP was expressed in HeLa cells stably expressing TEV protease under a doxycycline-inducible promoter, and the cells were treated with doxycycline, LSM14A-TEVP was cleaved, and the cleavage products were of the same size seen in enterovirus-infected cells. Unfortunately, we also saw slight cleavage in the absence of doxycycline, indicating some leakiness of the system, but the level of the

cleavage product substantially increased upon doxycycline treatment, and most importantly, it strongly correlated with the loss of LSM14A's ability to cause ISRE activation (Figure 7C)

2) In Fig. 6A/B, mutations of the P1' position on LSM14A does not prevent cleavage and in fact, leads to a larger cleaved C-terminal fragment protein. Is there another cryptic 2A site nearby? It is important to (i) show that the mutation does block 2A cleavage in an in vitro cleavage assay and (ii) to try and track down this other cleavage site. If you look at the immunoblots of LSM14A in Figures 2A and 3A, there is clearly another slower migrating band, suggesting another cleavage site. Even in It is important as all subsequent N-term and C-term LSM14A studies in Fig 7 are based on one of these cleavage sites. In Fig 7, is LSM14A cleaved under SeV infection and what is the status (immunoblotting) of expressing the N-term or C-term fragments in infected cells.
An interesting question is to monitor LSM14A localization (P bodies) by immunofluorescence. Perhaps LSM14A cleavage in infected cells disrupts its localization.

Excellent point! The loop region of LSM14A, which separates the N-terminal LSM14A domain from the C-terminal FGF boxes, and which is targeted by enteroviral proteases, contains a large number of FG, AG, VG, and MG motifs, each constituting a target sequence for enterovirus proteases. The data presented in Figure 5A shows that although diverse enteroviruses target the same region of the protein, they appear to cleave at different sites/motifs within this region. Therefore, the overarching result of the cleavage seems to be the separation of the N-terminal LSM14A domain of the protein from the C-terminal FGF boxes, rendering the protein non-functional in the context of ISRE activation. Since the second putative cleavage site was not revealed by our degradomic analysis, in the future studies, we will perform systematic mutagenesis to disrupt one target sequence at a time and examine its effect on virus-mediated cleavage.

The reviewer's point on LSM14A cleavage in SeV-infected cells is well taken. Since SeV is a negative-sense RNA virus with no encoded proteases, we doubt that the protein would be cleaved upon SeV infection. In fact, the data presented in Figures 5 and S9 show that infection of HeLa cells with another negative-sense RNA virus, vesicular stomatitis virus (VSV), did not result in cleavage of LSM14A or the other proteins cleaved by enteroviruses.

We agree with the reviewer that monitoring LSM14A localization in infected cells will be informative and plan to pursue this in future studies.

3) The role of LSM14A is a bit confusing. Fig 7D, RIG-I is required for LSM14A-dependent ISRE activation, however, in Fig 7F, SeV can still induce ISRE independent of LSM14A.

Could expression of the N- or C-term fragment of LSM14A have a dominant negative effect on RIG-I signaling? in CVB3-infected cells? Does LSM14A interact with RIG-I or other signaling factors? Answering these questions may provide specific insights into the functional role for cleavage of LSM14A.

We apologize for the ambiguity. Figure 7D shows that the presence of RIG-I is essential for LSM14A immune functions. However, as shown in Figure 7F, RIG-I doesn't need LSM14A for ISRE activation. Together this suggests that while RIG-I is indispensable for SeV-induced immune activation, LSM14A is not. LSM14A is a RIG-I cofactor, which when present, augments RIG-I signaling.

We did test the dominant-negative effect of N- and C-terminal fragments of LSM14A on ISRE activation but didn't observe any effect. We take the position that the other experiments proposed by the reviewer will be extremely useful in delineating the mechanism of action of LSM14A. However, we would like to emphasize that the primary objective of this study was to generate a comprehensive catalog of host proteins cleaved upon enterovirus infection. We then used LSM14A as a proof-of-concept to show the functional significance of one previously unknown virus-induced cleavage. In future studies, we plan to take systematic approaches, as proposed by the reviewer, to dissect the functional consequences and mechanistic details of LSM14A and other protein cleavages identified in the present study.

4) With the extensive analysis of the cleaved substrates, it would great to show the % of targets by viral protease (3C, 2A) vs other (cellular). How does this percentage change over the time course of infection?

Good point! We attempted to group protein cleavages into those mediated by virus vs host proteases, but the analyses was complicated by two factors: The target sequences of most of the host proteases are unknown, and secondly, our degradomic data and subsequent Western blots showed that enteroviral proteases can also cut proteins at non-canonical sites, for example, 2Apro cleaved ADAR1 at an “NA” motif. Therefore, unless the protein cleavages are validated by western blot and their cleavage site confirmed by mutagenesis, it is difficult to group them into virus vs host targets. Having said this, we did do the suggested analysis for caspase targets and found that their numbers increased with time, reaching their peak at late infection, when a large number of cells exhibited cytopathic effects (this information is provided on **P. 9, lines 179-184**).

5) P. 9, lines 88-90. This was a bit vague. It would be more informative to show the percent of proteins that showed the expected size. Although many targets were identified and validated to be cleaved under infection and in vitro, it is important to show that some of the cleavage sites identified are real and directly cleaved by 3C/2A. Mutation of the cleavage sites (within a tagged protein) should prevent cleavage in infected cells and in vitro. This was only attempted for

LSM14A. Alternatively, it is more direct to use purified, recombinant protein (can be bought) in in vitro cleavage assays.

We have now incorporated information on the percentage of proteins showing the expected cleavage pattern into the text (**P. 10, lines 215-217**). As we begin to explore the functional relevance of the identified proteins in future studies, we will generate mutants, as suggested by the reviewer, and confirm protein cleavage sites by various approaches.

6) There were several statements that were too strong "enterovirus-mediated cleavage renders LSM14A inactive". " enterovirus 2Apro-mediated cleavage blunted the ability of this protein to contribute to antiviral immune signaling." p. 17 " However, when LSM14A is cleaved by 2Apro, it can no longer mediate immune activation, indicating a loss of function."
These are not true, there is no evidence of this in enterovirus infected cells or results that directly show that 2Apro cleavage of LSM14A inactivates immune activation.

Thanks for pointing this out. In light of the reviewer's comments, we have now softened and improved the accuracy of these statements as follows:

P. 16, lines 346-347: "*These results clearly indicated that the enterovirus-mediated cleavage renders LSM14A non-functional*" has been replaced with, "*These results clearly indicated that the N- and C-terminal cleavage products of LSM14A do not cause ISRE activation upon SeV infection*". (**P.18, lines 372-373**)

P. 17, lines 382-383: "*enterovirus 2A^{pro}-mediated cleavage blunted the ability of this protein to contribute to antiviral immune signaling*" has been changed to, "*the truncated forms of this protein generated by enterovirus 2A^{pro}-mediated cleavage do not cause ISRE activation in response to SeV infection".* (**P. 19, lines 410-411**)

P. 18, lines 392-393: "*However, when LSM14A is cleaved by 2A^{pro}, it can no longer mediate immune activation, indicating a loss of function*" has been replaced with, "*However, when LSM14A is cleaved by 2A^{pro}, it can no longer mediate SeV-induced ISRE activation, indicating a loss of function*". (**P. 20, lines 418-419**)

Minor comments:
– Figure 1B - are the proteins grouped in a particular manner? More clarification is needed.

Figure 1B shows a binary heatmap of peptides, not proteins, with each row representing a unique Abu-labeled peptide. The peptides are not presented in any particular order. This is now clarified in the figure legend.

– Figure 6A - it would be good to have a cartoon above with the P1-P4/P1'-P4' amino acids to help understand how the mutations would disrupt cleavage.

Excellent suggestion! We have now included a cartoon showing P8-P1/P1'-P8' residues and highlighting the P1' amino acid that we mutated.

– P. 5, line 103. The N-terminomics approach cited in ref 21 is not similar but is distinct using a negative-selection approach whereas the subtiligase is a positive-selection.

Thanks! The phrase "*a similar N-terminomics approach*" has been changed to "*another N-terminomics approach*" to address the reviewer's point (**P. 6, line 117**).

– P. 8, line 61 - missing reference for cleavage site of caspases

The reference has been added (**P. 9, line 181**)

Referee #2 (Remarks to the Author):

In this manuscript, Saeed et al. identify sites of host protein cleavage upon enterovirus infection, validate a number of these sites orthogonally, and examine one proteolytic substrate in more detail. It is a substantive and thorough piece of work and perhaps long overdue for the field. It is worthy of publication. None of the following comments detract measurably from this conclusion.

The study raises interesting future questions about how 2A site specificity is achieved. It is also interesting to consider how infection may modulate the cell's natural peptidomic landscape via, for example, the TAP transporters (the MS portion of the study does not seem to require neo-N-termini to represent a simple bisection, or for peptides to be intracellular).

The initial portion of the study, namely the N-terminomics of CVB-3-infected HeLa extract via peptide ligation to unblocked N-termini, was likely challenging to perfect despite optimization: Here, the presence of a substantial unmodified proteome, even after the enrichment steps, is suggested by the setting of Abu as a variable modification in order to achieve acceptable FDR. In future work, this could perhaps be addressed by implementing an uninfected/infected isotope split. In this regard (P.6/Line 19), it is not clear what is meant by "multiplexed

samples” since it appears they were not multiplexed according to M&M.

The authors identify unique human targets from single peptides, and the latter would need to be of sufficient length and quality (of fragmentation spectra) to evince unique matches in the human proteome. In this regard, it is not clear whether a peptide length filter was imposed: Parental spectra scanned from a low m/z of 140 Th, sufficiently low to identify Abu-tagged peptides just 2 aa in length. The fragmentation method (presumably HCD, since it was a Q-E?) does not seem to be specified. The authors identify cleavage sites from the downstream portion of the cleavage site only. Albeit unlikely, is there an assumption data do not include secondary sites from exopeptidase nibbling?

In the following comments, note that the first digit of all line numbers was cut off on my pdf. Also, the supp data did not seem to be available for review (at least in the links/logins I could find). The authors fund these oversights by the journal.

Thanks for the excellent description and favorable review of our work.

1. We agree with the reviewer that the term “multiplexed samples” in this context is confusing. We have now replaced it with “multiple samples” (**P. 7, line 135**)

2. The reviewer has raised the important question about the peptide length filter. We indeed did impose the filter, considering only those peptides that were at least 7 amino acid long, which is also the default value in the search algorithms, such as Mascot and MaxQuant. As an additional note, the medium length of the matched peptides was 12, with 80% of all peptides being between 9 and 22 amino acids. We have now added this information to the M&M (**P. 35, line 744**).

3. The reviewer is correct in that we used HCD for ion fragmentation. We have now mentioned it in the M&M (**P. 34, line 734**).

4. To account for exopeptidase nibbling (a.k.a ragging), we wrote, validated, and implemented a script in R allowing for removal of those peptides from our final analysis that presumably arose from exopeptidase activity. To further explain this, since enteroviral proteases preferentially, almost exclusively, target G and A at the P1’ position, we removed from our analysis those peptides that had their N-terminal amino acid 1-3 positions downstream of a G or A, but where the full-length, presumably authentic, peptide starting with actual G or A was also detected. This information has now been added to M&M section under the “Bioinformatics analysis” heading (**P. 35, line 761-764**).

SPECIFIC COMMENTS:

1) P.8/Line x72: Were the known protein targets confirmed at the level of target site within the protein?

We have now confirmed the known targets at the level of the cleavage site and added the following sentence to the text to reflect our finding: *"Most importantly, the peptides for eIF4G, HNRPD, HNRPM, MAVS, and PABP corresponded to the known cleavage sites in these proteins, although additional peptides were also detected."* (**P. 9-10, lines 196-198**)

2) P.9 Line x80 vs. line x84: Was it 81 or 90 proteins?

We apologize for the confusion. The information provided in line 184 is changed to make it consistent with the numbers given in line 180 (now **P. 10, lines 205 and 208-212**)

3) P.19/Line x91: How do the authors suppose that enteroviral proteases target intercellular junctions?

Our degradomic dataset contained some of the intercellular junction proteins, such as PANX1, which is a gap junction component. However, since this set of proteins did not emerge as a predominant group in our pathway analysis, we have now replaced "Intercellular junctions" with "cytoplasmic ribonucleoprotein granules" based on the finding that several resident proteins of these granules, such as LSM14A, DDX6, DCP1A, FAM120A, and MATR3, were among the list of protease substrates. Also, our computational analyses showed that ribonucleoprotein complexes were one of the major targets of enterovirus-mediated proteolysis (Figure 4C).

In Fig. 2A, might virus-induced loss of function/gain of function correspond to quantitative cleavage/incomplete cleavage respectively? Is it clear why cleavages can be detected prior to the first appearance of eGFP?

Interesting point—something we have also wondered about! In future studies, we plan to perform systematic mutagenesis of the cleavage site(s) with the goal to find a correlation between the efficiency of cleavage and the resulting loss/gain of function.

Some host proteins, particularly eIF4G and NUP98, seem to be extremely sensitive to enteroviral $2A^{pro}$. Based on our unpublished observations, the amount of protease generated from the translation of the incoming viral RNA appears to be sufficient for eIF4G cleavage, while eGFP becomes detectable later, only after the virus replication kicks in.

P.28/line 601: Induction temperature seems missing.

Thank you for spotting this! The induction was carried out at 28ºC. We have now added this information to the M&M section (**P. 32, line 682**).

P.29 line 623: 150 microG/microL -> 15 *µ*g/*µ*l

Thanks! Corrected (**P. 33, line 704**).

LC-MS/MS section of M&M should specify nanocolumn dimensions/packing/details, along with high m/z (scan range) and fragmentation method. "tandem MS experiments" -> "Tandem MS".

We apologize that the information regarding nanocolumns was lost in edits. We have now added the following sentence to the M&M section: *"For reversed-phase liquid chromatography (LC), we used* C_{18} *columns of 12 cm length and 75 µm internal diameter packed with beads of 3 µm particle size (Nikkyo Technologies, Japan)."* (**P.34, lines 728-730**)

The m/z scan range is difficult to specify, because it varies from precursor to precursor. In other words, we had set the lowest m/z value at 140, but the highest m/z value was calculated by the mass spectrometer based on individual precursors. For example, for a doubly charged ion at m/z 700, the maximum range will be around m/z 1,500 (m/z 2x700 plus margin).

We used HCD for ion fragmentation and have added this information to the manuscript (**P.34, line 734**).

"Tandem MS experiments" has been replaced with "Tandem MS" (**P. 34, line 737**)

The authors could have perhaps included a sentence, somewhere, on the known functions of p-bodies. (e.g. in cytoplasmic mRNA turnover).

Thanks for this suggestion! We have now included this information in the Results section as follows: *"LSM14A mainly resides in p bodies, cytoplasmic ribonucleoprotein granules involved in mRNA turnover, and has been previously implicated in antiviral immunity against some DNA and RNA viruses."* (**P. 16, lines 339-340**)

Wording:

P.4/Line x78 ("2A is only responsible for its own cleavage from the upstream

structural protein") should perhaps be re-worded given 2A's diverse set of cellular substrates.

We have added the phrase, "*in the context of viral polyprotein processing*" at the beginning of the sentence to make it clear that the role of $2A^{pro}$ described in this sentence is strictly in reference to its contribution to the viral polyprotein processing (**P. 4-5, lines 88-89**).

P.7 Lines x34/x35: "Intensity of labeling" could perhaps be replaced with "Diversity of labeling".

We agree. The word "intensity of labeling" in this context is misleading. So, we have replaced it with "magnitude of labeling" (**P. 7, line 152**)

P.3/Line x66: Close bracket.

Good catch! Corrected (now **P. 4, line 74**).

P.2/Line x44: Add "a". P.9/Line 81: were -> are.

Both fixed.

P.14/Line x99: "Showed" -> "indicated"

Changed (now **P. 16, line 341**).

P.17/line x47 "viruses" -> "enteroviruses" (since VEEV & SeV seem not to).

Valid point! Corrected (now **P. 19, line 395**).

P.17/line x51 "Novel proteins" -> "novel substrates".

Corrected (now **P. 19, line 399**).

P.18/Line x70 "Two C-terminal domains" -> "Two domains C-terminal to the unstructured region".

We have modified this sentence as follows: *"LSM14A is a 463 aa protein with an N-terminal LSm domain of 76 aa (aa 1-76) followed by an intrinsically unstructured region of around 200 aa and **then** two C-terminal motifs, namely a DFDF box (aa 291-316) and an FDF_TFG box (aa 361-397)."* (**P. 20, lines 419-422**)

Referee #3 (Remarks to the Author):

This manuscript describes the first attempted comprehensive examination of the extent and breadth of human proteins cleaved during infection by the two enterovirus proteinases. The authors employed technical strategies based on a bioengineered protein ligase, subtiligase, to label and capture nascent C-terminal protein cleavage fragments which were identified by LC-MS/MS.

This work indirectly builds on prior findings by others using similar strategies to ID cleavage products of poliovirus and CVB3 3Cpro generated in vitro. This manuscript goes further by using similar technical approaches to catalogue cleavages by both virus proteases in virus infections with five different enteroviruses (PV, EVD70, EVA71, HRVA16, CVB3). Several identified protease cleavages were validated by expression of proteases in cells and immunoblot approaches to examine protein cleavage. Other validation included in vitro cleavage with purified proteinases to confirm 38 targets. The authors also compared cleavage products generated in Caco2, RD, SK-N-SH cells with Hela cell cleavage products, which confirmed similar cleavage across cell types. Other findings presented are the exact cleavage sites targeted by each virus proteinase, which mostly confirmed the previous known cleavage site specificity of each proteinase but revealed greater substrate recognition variability, particularly with 2Apro. These exceptions to known cleavage site requirements were noted and are not unexpected. There is no mention of previously documented conformational constraints that also govern substrate specificity.

The authors focused more on one newly described cleavage target LSM14a, a factor in P-bodies with a role in innate immunity. LSM14a was found to be cleaved by 2Apro, a series of experiments indicated some functional impact of this cleavage in disrupting innate immune activation. This included engineering an TEV protease cleavage site where 2Apro cleaves to demonstrate that cleavage at the same site in LSM14a with a different protease disrupted its function in transcriptional activation of ISRE. The authors then further pursue the mechanisms of LSM14a activation of innate responses, performing experiments with knockdown of MAVS, RIG-I, STAT1. These experiments, clarified, but did not reveal the true function of LSM14a, indicating LSM14a is not essential for RIG-I activation but may serve as a co-factor.

Overall the manuscript describes a large amount of work and resulting data that provides a useful catalog of viral proteinase targets, and shows most are conserved between viruses but not all. Ultimately, many of the new targets, perhaps most, will represent noise, by-stander targets that may have no real impact on virus replication advantages. This is revealed in the breadth of cell pathways of the proteins identified. However, some new protease targets could reveal new mechanisms of import in virus pathogenesis. However, the authors

note that in toto, the net results from multiple virus cleavage targets further reduce host gene expression beyond the well-known virus shutdown of translation and transcription. The repository of information here may be of general interest to virologists in other systems. Other than the increase in the catalog of proteins known to be targeted by enteroviruses, there are few findings concerning enterovirus pathogenesis beyond the LSM14a results presented.

Thanks for the encouraging remarks—much appreciated.
